# Antiepileptic drug-induced hypersensitivity syndrome with liver function abnormality and fever as the first manifestation: A case report

**DOI:** 10.1097/MD.0000000000032657

**Published:** 2023-01-20

**Authors:** Mei Liu, Xin-Yu Ci, Han Huang, Mei-Juan Zhang

**Affiliations:** a Department of Gastroenterology, Shandong Provincial Qianfoshan Hospital, Shandong First Medical University, Jinan, Shandong, China; b Department of Gastroenterology, The First Affiliated Hospital of Shandong First Medical University & Shandong Provincial Qianfoshan Hospital, Jinan, Shandong, China; c Department of Health Management, The First Affiliated Hospital of Shandong First Medical University & Shandong Provincial Qianfoshan Hospital, Shandong Engineering Laboratory for Health Management, Jinan, Shandong, China.

**Keywords:** diagnosis, DRESS syndrome, therapy

## Abstract

Drug reaction with eosinophilia and systemic symptoms (DRESS) syndrome is a severe cutaneous drug reaction characterized by fever, lymphadenopathy, hematologic abnormalities, single or multiple organ involvement, and viral reactivation.^[1]^ The most frequently reported offending drugs are aromatic antiepileptic agents, antibiotics, and allopurinol.^[2]^ Though a relatively rare syndrome, DRESS can lead to severe multi-organ system dysfunction, and in some cases even death. DRESS is one of the severe drug eruptions in dermatological diseases, but it is difficult to diagnose for internist. In this paper, a typical drug hypersensitivity syndrome with abnormal liver function and fever as the first manifestations was reported. The objective of this study was to improve the understanding of rare drug hypersensitivity syndrome in digestion and other fields, and to avoid missed diagnosis and misdiagnosis.

**Patient Concerns:**

A 33-year-old Chinese female was initially diagnosed with acute hepatic insufficiency. Combined with the suspicious drug history, she developed DRESS with fever, target erythema, left lymph node enlargement, hematological abnormalities, and abnormal liver function.

**Diagnoses:**

Combined with the above characteristics, liver toxicity is the main manifestation, accompanied by fever, mainly moderate to high fever (above 38 °C) , sporadic rash, other organs (kidney, immune system) damage, and a marked increase in eosinophil granulocytic. Therefore the patient was diagnosed with definite case of DRESS syndrome based on clinical and laboratory findings.

**Interventions:**

Hormones (methylprednisolone 60 mg/day for 12 days and 80 mg/day for 12 days) and immunoglobulins (intravenous immunoglobulin 10 g/day for 5 days and 20 g/day for 7 days) were given.

**Outcomes:**

The patient was discharged from the hospital after recovery. One month after discharge, she was re-admitted to the hospital because of elevated blood sugar and was diagnosed as diabetes.

**Lessons:**

DRESS syndrome is a rare but life-threatening hypersensitivity reaction. The mortality will be very high if it’s not diagnosed and treated timely. This paper presents a successful case of methylprednisolone plus intravenous immunoglobulin therapy, which provides a stronger evidence for the future diagnosis and treatment of the disease.

## 1. Introduction

Drug reaction with eosinophilia and systemic symptoms (DRESS) syndrome is a severe adverse drug reaction characterized by an eruption, fever, lymph node enlargement, hematologic abnormalities, multiorgan involvement and viral reactivation,^[1]^ especially human herpesvirus-6 but also cytomegalovirus, and Epstein–Barr virus (EBV). The eruption in DRESS can present in different forms of expression and the most common types are polymorphous maculopapular eruption and facial edema. Furthermore, patients also present with pustules, purpura, infiltrated plaques, etc.^[3]^ Hematologic abnormalities may include eosinophilia, thrombocytopenia, and atypical lymphocytosis.^[4]^ Besides eruption and hematologic abnormalities, the other most commonly involved organs are liver, kidney, and lungs. The cardiovascular system, other gastrointestinal organs, skeletal musculature, and the nervous system can also be involved as well. Therefore DRESS is a rare but potentially life-threatening systemic disease.^[5]^

The most frequently reported offending drugs inducing DRESS are aromatic antiepileptic agents, antibiotics, and allopurinol.^[2]^ Timely diagnosis and rapid cessation of causative drugs are critical for patients.^[1]^ Treatment includes systemic corticosteroid and intravenous immunoglobulin (IVIG), or even a combination of both. In addition, symptomatic treatment is essential, such as anti-infection, hepatoprotective, anti-allergic, etc.

## 2. Case presentations

A 33-year-old woman was admitted to the emergency department with the complaints of high grade fever, weakness, generalized xanthochromia, and erythematous eruption. Phenytoin sodium and levetiracetam had been added 1 month before the admission. Symptoms of high fever began to appear 1 week before the admission, so the medication was stopped. The body temperature was up to 39.3°C, mostly in the afternoon and at night, while it dropped to normal after using the “indomethacin” and rose again the next day, with nausea, vomiting, diarrhea, and watery stools. She once took “Gankang” on her own and went to the local clinic for treatment, which was ineffective. A red rash appeared on the head 5 days before the admission, which then spread throughout the body with intermittent itching. On presentation to local hospital, she had worsening leukocytosis, which was diagnosed as “gastroenteritis” and given “levofloxacin” and other treatment for 1 day, and the symptoms improved slightly. After repeated symptoms and poor treatment effect, she was treated in the emergency department of our hospital last night.

**Figure 1.**
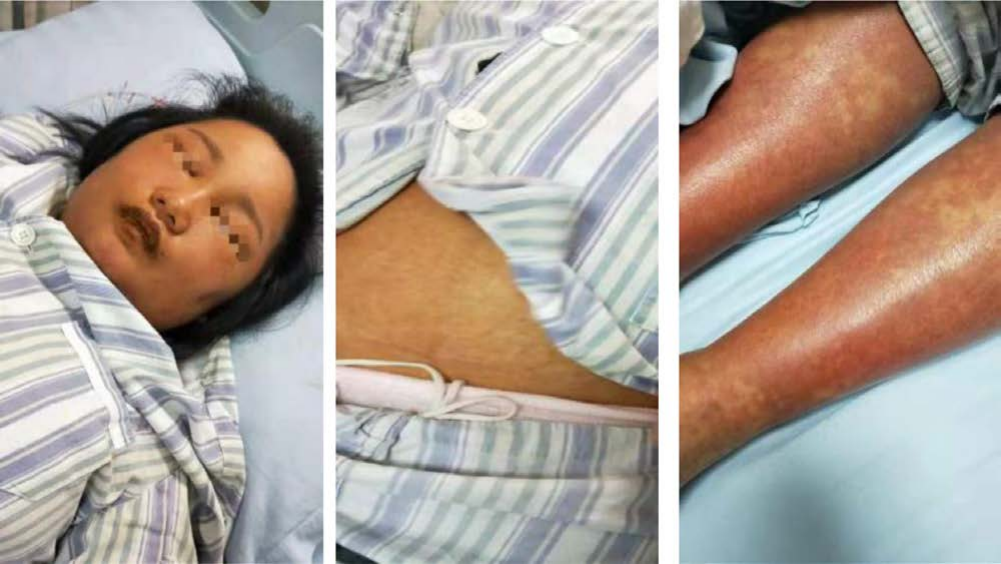
Facial edema, generalized xanthochromia and target shield symptom.

On examination, physical findings were as follows: temperature, 39.0°C, pulse rate, 109 bpm, respiration, 23 breaths/min, and blood pressure, 113/66 mm Hg. Skin showed generalized xanthochromia and target erythema which was widespread on trunk and 4 limbs (Fig. 1). Left enlarged cervical lymph nodes were determined. Furthermore, she had sclera icterus and facial edema. Physical examination revealed no splenomegaly and hepatomegaly.

Laboratory findings revealed hemoglobin (133.0 g/dL) and leucocyte (25.18 × 10^9^/L) counts with a differential of 71.00% neutrophils, 5% lymphocytes, and 13% eosinophils. Eosinophils number was 3.273 × 10^9^/L. Prothrombin time, international normalized ratio, D-dimmer and fibrin degradation product were elevated at 16.5 s, 1.42, 8.76 mg/L and 26.08 mg/L, while prothrombin activity was degraded at 51.8%. Aspartate aminotransferase, alanine aminotransferase, alkaline phosphatase, and γ-glutamyl transferase were elevated at 369.0, 429.0, 358.0, 612.0 U/L, respectively. Total bilirubin and direct bilirubin were also elevated at 117.2, 78.7 umol/L, respectively. C-reactive protein was elevated at 58.1 mg/dL (normal range < 5 mg/L). Procalcitonin was elevated at 1.037 ng/mL (normal range < 0.05 ng/mL). Erythrocyte sedimentation rate was normal. Glycosylated hemoglobin was normal. Virological examinations for hepatitis A, B, C, and E, the EBV, parvovirus B-19, rubella, measles, toxoplasmosis, typhoid fever, paratyphoid fever, epidemic hemorrhagic fever, brucellosis, bunia virus, human herpes virus, cytomegalovirus, and herpes simplex virus were negative. Ultrasonography revealed hepatomegaly.

**Figure 2.**
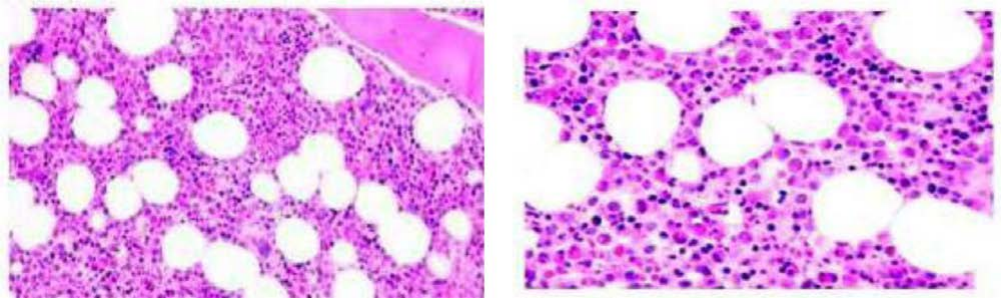
Pathologic diagnosis: Partial hyperplasia of the bone marrow is more active, and partial hyperplasia is extremely active: the ratio of granules and reds increases, and granular hyperplasia is obviously dominant, MPO (more+); Cells of all stages of granule and erythroid can be seen, and cells in the naïve stage are mildly hyperplasia, CD34 and CD117 (individual+), and eosinophils are obviously hyperplasia; monocytes did not see significant hyperplasia, lysozyme (partial+), partially mature megakaryocytes scattered in distribution, CD61(+), no hemosiderosis and fibrous tissue hyperplasia, reticulated fiber staining: (0–1+). Images consistent with eosinophilic hyperplasia disease are recommended in conjunction with clinical and other laboratory tests. MPO = Myeloperoxidase.

After admission, the bilirubin level continued to rise. Besides there was a tendency to become more severe liver disease, accompanied by allergic reactions (fever with rash and eosinophils significantly increased), and the patient’s laboratory tests suggested the presence of infection, so the following treatment was given. The patient got treated with biapenem and yanhuning for anti-infection, with ganciclovir for antiviral therapy, with ebastine, cetirizine and calamine lotion for anti-allergic, with magnesium isoglycyrrhizate, tiopronin and hepatocyte growth promoting factor for liver protection, with butadilfonyl sulfonate adenosine for cholagogic, with alprostadil for improve circulation, with lansoprazole for acid inhibition, with L-glutamine and sodium gualenate for the protection of gastric mucosal and nutritional support therapy. However, symptoms of high fever persisted after the above treatment. Reexamination tests revealed worsening leukocytosis, significantly elevating eosinophils, lymphocytes, and monocytes (Figs. 1 and 2). Thus, the patient was suspected of leukemoid reaction caused by severe infection.

In order to exclude hematologic diseases such as leukemia and lymphoma, bone marrow puncture and ultrasound-guided biopsy of left cervical lymph nodes were performed. Bone marrow flow cytology showed that the proportion of myeloid protocells was not high, and no obvious abnormalities were seen in the phenotype. The granulocytes were dominated by myelocytes and promyelocytes, and no obvious abnormalities were seen in the phenotype, and a large number of eosinophils were seen. The proportion of plasma cells to nucleated cells was 1.95%. No abnormal phenotypes were seen in mononuclear, erythroid, and lymphocytes. Bone marrow pathology showed the same result (Fig. 2). Lymph node biopsy revealed a small amount of lymphoid tissue (neck), fibrous tissue, and striated muscle tissue, with more infiltration of eosinophil granulocytic and neutrophil.

The main problem of the patient during diagnosis and treatment was obvious abnormal liver function, fever, and hematological abnormalities, so relevant examinations were constantly improved. First, we conducted a comprehensive investigation for the cause of liver function abnormalities. Excluding abnormal liver function caused by common factors such as viruses, autoimmunity, alcohol, and genetic metabolism, combined with the suspicious drug history, drug factors were considered. Second, from out-of-hospital onset to admission, the patient had recurrent fever and underwent comprehensive screening for the cause of fever, including lung computed tomography imaging and hematology tests such as brucellus cloglutination test, anti-cytomegalovirus-IgM, anti-EBV-IgM, and emission computed tomography, which showed a serious infection. Third, we perfected the tests to rule out hematologic abnormalities, including bone marrow aspiration and ultrasound-guided lymph node biopsy, and hematologic abnormalities were considered.

Combined with the above characteristics, drug-induced liver injury is the main manifestation, accompanied by fever, mainly moderate to high fever (above 38 °C) , sporadic rash, other organs (kidney, immune system) damage, and a marked increase in eosinophil granulocytic. Therefore the patient was diagnosed with definite case of DRESS syndrome based on clinical and laboratory findings (Table 1).

**Table 1:** DRESS (RegiSCAR).

1. Acute rash
2. Reaction suspected drug-related
3. Hospitalization
4. Fever (>38°C)
5. Laboratory abnormalities (at least 1 present)
a. Lymphocyte above or below normal
b. Low platelet
c. Eosinophilia
6. Involvement of >1 internal organ
7. Enlarged lymph nodes >2 sites

The first 3 criteria are necessary for diagnosis, and the presence of 3 out of the other 4.

DRESS = drug reaction with eosinophilia and systemic symptoms, RegiSCAR = Registry of Severe Cutaneous Adverse Reactions.

**Figure 3.**
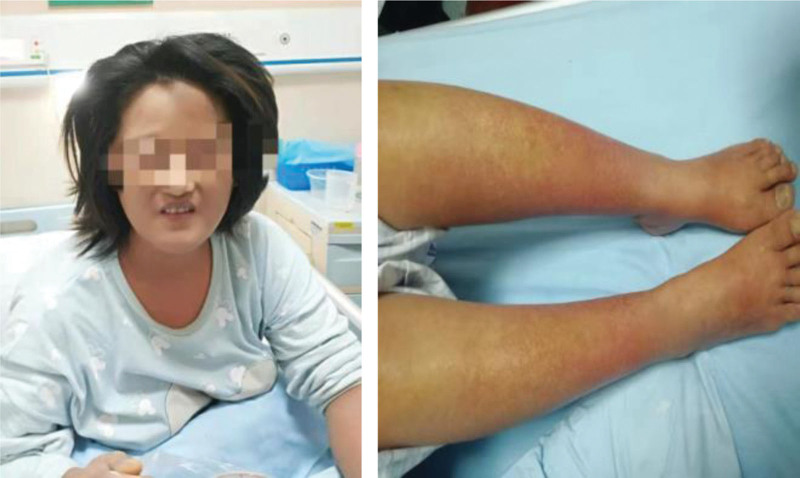
The patient’s skin symptoms improved significantly.

**Figure 4.**
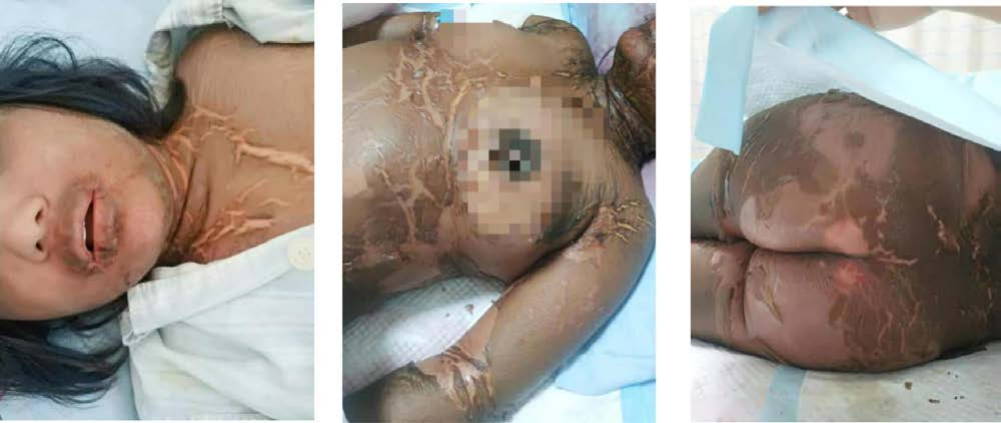
Diffuse exfoliating morbilliform rash.

**Figure 5.**
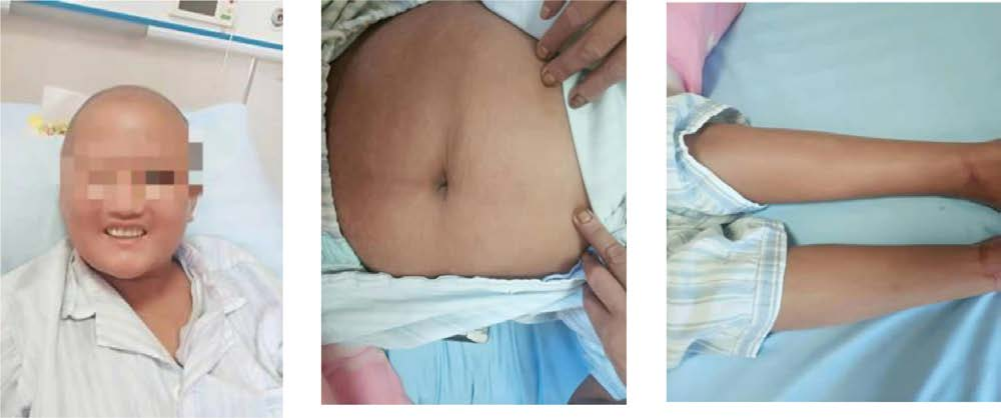
The rash and edema all subsided.

**Figure 6.**
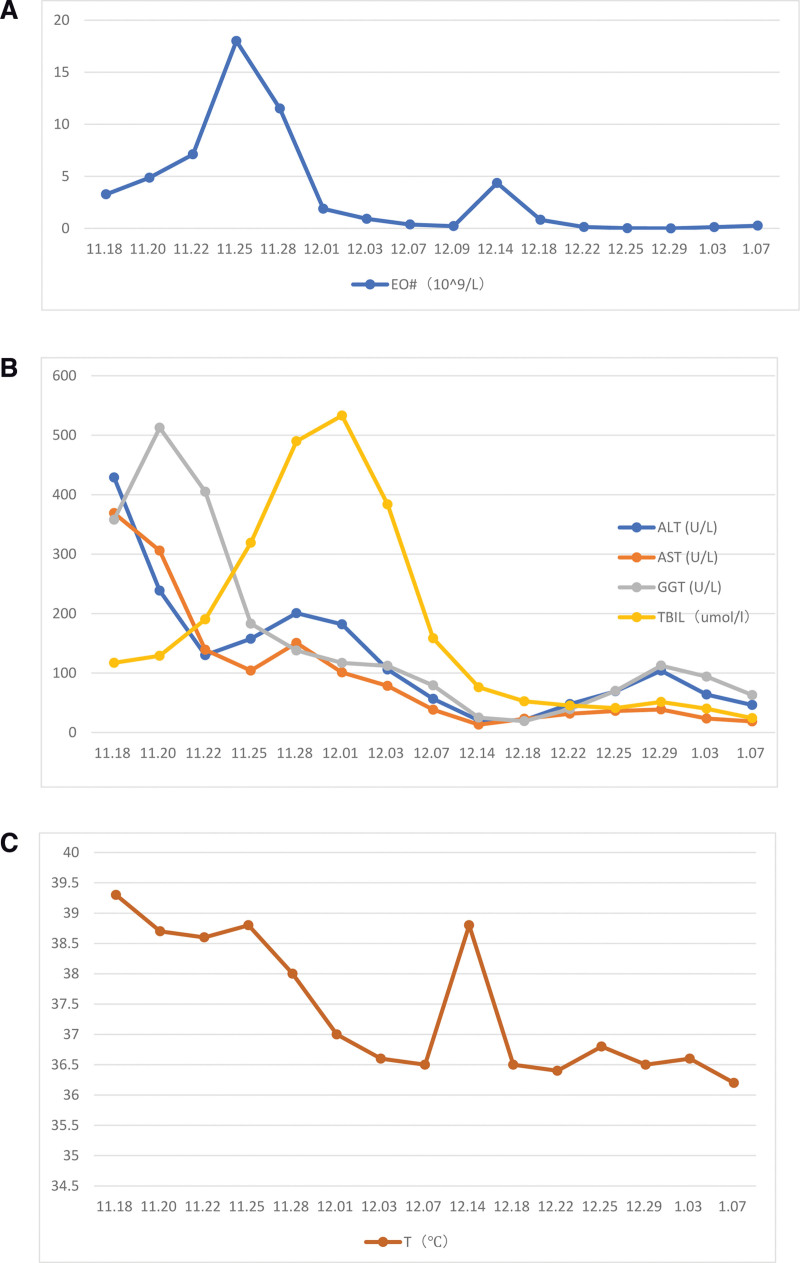
Change curve of (A) eosinophil count, (B) different liver function index, and (C) temperature.

Based on the principles of treatment of the disease, hormones and immunoglobulins were given. Methylprednisolone was given at a dose of 60 mg/d for 12 days and IVIG was given at a dose of 10 g/d for 5 days, in the meanwhile, anti-infection, hepatoprotective, enzyme-lowering, acid suppression, anti-allergic, and symptomatic supportive therapy were also given. After treatment, the patient’s symptoms improved significantly (Fig. 3), and there was no fever again. The results of the reexamination of complete blood count and liver function were significantly improved compared with before. Then the dose of methylprednisolone was adjusted to 40 mg/d. However, after the adjustment, the patient’s condition worsened with renewed fever, rash, edema of skin tissue, and diffuse scales throughout the body (Fig. 4). Therefore, the treatment regimen was adjusted again. Methylprednisolone was given at a dose of 80 mg/d for 12 days and IVIG was given at a dose of 20 g/d for 7 days, while other treatments remained unchanged. After comprehensive treatment, the rash and edema of the patient significantly improved and even disappeared (Fig. 5). Furthermore, the laboratory findings showed the count of eosinophil (Fig. 6A), the values of transaminases (Fig. 6B) and total bilirubin (Fig. 6B) declined slowly throughout the duration of treatment, which means the improvement of her disease. In addition, the body temperature gradually stabilized (Fig. 6C), after her condition was stable and did not recur, she was discharged from the hospital after being changed to oral hormones.

After discharge, the patient took the drug regularly and reviewed DRESS syndrome regularly. Her condition was stable and did not recur. One month later, due to the rise in blood sugar, she visited our hospital again and was diagnosed with diabetes which was considered to be long-term sequelae of DRSS syndrome because there was no previous history of diabetes mellitus.

## 3. Discussion

DRESS syndrome is a severe adverse drug reaction characterized by skin rash, fever, lymph node enlargement, hematologic abnormalities, multiorgan involvement, and viral reactivation. Indeed, the skin rash is often polymorphic and can include maculopapular exanthema and lichenoid, exfoliative manifestations, etc.^[6]^ Besides, in some severe cases, there may be present target lesions with erythema multiforme-like skin manifestation, which usually is accompanied by significant dermal edema and inflammation,^[7]^ as congruent with our cases. Organs involved mostly include liver, kidney, and lung, and the most common manifestation is abnormal liver function, which is presented in our case. Besides acute sequelae, patients with DRESS are also at risk of long-term systemic autoimmune sequelae, which can appear months after resolution of the cutaneous eruption and acute systemic involvement.^[1]^ As our case shows, the patient developed elevated blood sugar and was diagnosed with diabetes within a month of discharge.

The most commonly used diagnostic criteria of DRESS are formulated in Europe and Japan. To diagnose DRESS cases, the Registry of Severe Cutaneous Adverse Reactions (RegiSCAR) consortium established its criteria in 2007 (Table 1).^[7]^ Besides, there is also a scoring system of RegiSCAR (Table 2).^[8]^

**Table 2:** Scoring system for classifying the cases of DRESS as possible, probable, or definite.

Scores	No	Yes	Unknown
Fever (≥38.5°C)	−1	0	−1
Enlarged lymph nodes (≥sites, ≥1 cm)	0	1	0
Atypical lymphocytes	0	1	0
Eosinophilia, cells/µL	0		0
700–1499 or 10%–19.9%		1	
≥1500 or ≥20%		2	
Skin rash	0		0
Extent: >50%	0	1	0
At least two of these: edema, infiltration, purpura, scaling	−1	1	0
Biopsy suggesting DRESS	−1	0	0
Internal organ involved	0		0
One		1	
Two or more		2	
Resolution in ≥15 days	−1	0	−1
At least three biological investigations performed and negative to exclude alternative diagnosis	0	1	0

Final score: ≥2 indicates no case; 2–3 indicates possible case; 4–5 indicates probable case; ≥5 indicates definite case.

DRESS = drug reaction with eosinophilia and systemic symptoms.

The presented patient was awarded 5 points according to RegiSCAR, indicating the following: eosinophilia of peripheral blood = 13% (1 point), target erythema which was widespread on trunk and 4 limbs and facial edema (2 points), involvement of internal organs (hepatomegaly and impaired liver function) (1 point), and timing (occurrence of the first symptoms 23 days after the treatment of antiepileptic), as well as the exclusion of another, alternative diagnosis (1 point). Furthermore, our case also complied with the diagnostic criteria in Table 1.

At present, there are no evidence-based management guidelines and prospective clinical trials to guide the treatment of DRESS, and all recommendations are based on case reports^[9,10]^ and expert opinion. The first principle of treatment of DRESS syndrome is to identify and discontinue the causative drug.^[9]^ In this report, our patient ceased the phenytoin sodium and levetiracetam when symptoms of high fever began to appear. Treatment includes topical and systemic immunomodulatory therapy, and treatment of various complications. The optimum treatment is systemic corticosteroids, but it may increase the risk of complications such as opportunistic infections.^[10]^ Therefore, changes in infection indicators need to be closely monitored during treatment. The recommended dose is 40 to 60 mg/d,^[11]^ which is needed to gradually reduce over a period of 10 weeks to prevent the excessive suppression of immune response to various pathogens.^[11]^ Besides, topical corticosteroids are part of the management of DRESS. In addition to corticosteroids, IVIG was also reported to be used for DRESS syndrome treatment.^[5]^ In this report, we gave the patient the treatment of methylprednisolone plus IVIG after a definitive diagnosis, which is proved to be efficient.

Patients with DRESS are also at risk for long term systemic autoimmune sequelae, which can be divided into 2 main categories for people of different ages. For young people, we usually consider autoimmune sequelae such as diabetes, autoimmune thyroiditis, autoimmune hemolytic anemia, etc. For elderly patients, we should pay more attention to the vital organ failure. Therefore we need to monitor even after clinical resolution of the acute phase of DRESS. Our patient acquired secondary diabetes. Approximately 3 months after the drug hypersensitivity episode, she experienced the onset of polydipsia and polyuria. At this time, her blood glucose concentration was 32.41 mg/dL and her blood was also positive for ketone bodies as determined by a serologic test. Her glycosylated hemoglobin was 9.6% and a random C-peptide was normal. Serum glutamic acid decarboxylase antibodies were detected. A diagnosis of secondary diabetes was made. After receiving standard treatment for ketoacidosis, the patient was discharged with a prescription for subcutaneous insulin and oral hypoglycemic agents. Her blood glucose concentrations have been controlled well by long-term insulin injections for the past 2 years. Diabetes is one of the common long term sequelae, whose specific pathogenesis is not clear by now. However, there have been reported that glucocorticoids (GCs) can raise blood glucose levels in both diabetics and non-diabetics. The predominant mechanism responsible for glucose intolerance after administration of GCs is reduced insulin sensitivity. And report also suggests a more prolonged corticosteroid effect on insulin resistance in comparison with prednisone.^[12]^ Due to the application of corticosteroid, it’s still unclear whether the increased blood sugar levels is caused by the disease itself or the use of GCs.

What needs to be learned from this case lies in the following aspects. The first is about the diagnosis of the disease. As an internist, due to the lack of understanding of DRESS disease, we rarely consider DRESS when facing patients with abnormal liver function and hematological abnormalities. It is easy to miss diagnose or even misdiagnose, which may delay the patient’s condition. Therefore, DRESS should be considered timely when patients have recurrent fever, generalized rash, accompanied by abnormal liver function, and hematological abnormalities. The second is the treatment. The optimum treatment is systemic corticosteroids. This paper presents a successful case of methylprednisolone plus IVIG therapy, which includes dosage, efficacy, and duration of treatment. However, because there is currently no clear treatment guideline, the specific hormone dosage and reduction time still need to be analyzed on a case-by-case basis. In this case, due to the inappropriate time of dose reduction, the patient’s condition deteriorated again during treatment. Since then, we have adjusted the hormone dosage in time, and the patient’s condition has gradually improved. It is hoped that by summarizing the lessons learned this time, it will provide some help for the diagnosis and treatment of DRESS in the future. Finally, rash as one of the typical symptoms of DRESS, its manifestations are diverse, such as peeling, etc. Besides causing itching discomfort, infection and even sepsis, it also brings a psychological burden to the patients because of affecting the patient’s appearance, which may lead to anxiety, depression, and even affecting the progress of the diagnosis and treatment of the disease. Therefore, in addition to drug treatment, it is more necessary to give patients support of mental and psychological aspects.

## 4. Conclusion

DRESS syndrome is a rare but life-threatening hypersensitivity reaction. It’s essential to timely diagnose the disease and discontinue the causative drug. This paper presents a successful case of methylprednisolone plus IVIG therapy, which provides a stronger evidence for the future diagnosis and treatment of the disease.

## Author contributions

**Data curation:** Han Huang.

**Supervision:** Mei-Juan Zhang.

**Writing – original draft:** Mei Liu.

**Writing – review & editing:** Xin-Yu Ci.
